# Acute-Phase Inflammatory Response in Idiopathic Sudden Deafness: Pathogenic Implications

**DOI:** 10.1155/2012/216592

**Published:** 2012-11-06

**Authors:** Miguel A. López-González, Antonio Abrante, Carmen López-Lorente, Antonio Gómez, Emilio Domínguez, Francisco Esteban

**Affiliations:** ^1^UGC Otorhinolaryngology, Virgen del Rocio University Hospital, C/Manuel Siurot s/n, 41013 Seville, Spain; ^2^Faculty of Medicine, University of Seville, C/Doctor Fedriani s/n, 41009 Seville, Spain

## Abstract

The acute-phase inflammatory response in the peripheral bloodstream can be an expression of transient cerebral ischaemia in idiopathic sudden deafness. For this, a neurological and otorhinolaryngological examination of each patient, performing tests on audiometry, and tympanometry, haemogram, and cranial magnetic resonance were performed. The acute-phase inflammatory response manifests as an increased neutrophil/lymphocyte ratio that is detected 48–72 hours after the appearance of sudden deafness. This study shows that there is an acute-phase response in the peripheral bloodstream with an increased neutrophil/lymphocyte ratio as an expression of an inflammatory process that can be caused by transient cerebral ischaemia in sudden deafness. In addition, the increased neutrophil/lymphocyte ratio can rule out a viral origin of sudden deafness, since a viral infection lowers the neutrophil count and increases the lymphocyte count, thus reducing the neutrophil/lymphocyte ratio. These findings aid in understanding the pathogenic mechanisms involved in sudden deafness and offer better treatment to the patient.

## 1. Introduction

Sudden deafness is usually unilateral and tends to be associated with vertigo and tinnitus. The origin of this condition remains unknown for the majority of cases, although several infectious, vascular, and immune causes have been proposed. A careful examination of the patient is required in order to exclude treatable causes and life-threatening situations such as vascular processes and tumour diseases. Approximately, half of all patients have complete recovery within two weeks. Several different treatments have been used, including corticosteroids, antiviral drugs, rheology, and oxygen therapy. Although no single treatment has demonstrated decisive results, a short schedule of oral corticosteroids is generally recommended [1].

Within the spectrum of possible causes of sudden deafness, vascular processes could be candidates for the pathogenesis of this condition because of their acute/abrupt nature and the audiovestibular symptoms (deafness, vertigo, tinnitus, sensation of ear fullness, and hyperacusis) that occur in ischaemia of the inner ear [2–5]. However, this has not been conclusively demonstrated. In a similar manner, an ischaemic process would explain the spontaneous recovery observed in over half of all cases [6], once vascular reperfusion is achieved.

Cerebral ischaemia, even the transient one, is characterised by an acute inflammatory response in the ischaemic area, which, in turn, produces an acute-phase response in the peripheral bloodstream, where polymorphonuclear cell concentrations are increased in an attempt to combat the damage caused by blood deficiency [7–10]. This study attempts to describe the peripheral acute-phase inflammatory response associated with the appearance of idiopathic sudden deafness. There is growing evidence of the association of an inflammatory process with sudden deafness. Inflammatory cytokines such as interleukin-1*α* [11], interleukin-1*β* [12], interleukin 4 [13], interleukin 6 [14, 15], and matrix metalloproteinase-1 [16], as well as an increased neutrophil count in peripheral blood [15]. There is also indirect evidence such as being improved after treatment with local hypothermia [17] or worsening with vertebrobasilar flow decrease [18].

## 2. Materials and Methods

### 2.1. Single Cohort, Descriptive, Observational Study

#### 2.1.1. Patients

Fifty-Four patients were cared for in the emergency department due to sudden hearing loss during 2011. Inclusion criteria were the occurrence of sudden deafness in one ear of 30 db or more in at least three consecutive frequencies. Also showed symptoms of dizziness, aural fullness, tinnitus, and hyperacusis. No patient was taking steroid therapy.

#### 2.1.2. General Demographic, Clinical, and Audiological Parameters

55% of the patients were women and 45% were men. The mean age of the patients was 45.4 years, with a range of 24–78 years. The mean time of evolution between the appearance of the sudden deafness and the patient seeking medical care with the application of treatment was 6.4 days. The sudden deafness was unilateral in all cases: the right ear in 47% and the left ear in 53%. The emergency audiometric analysis revealed moderate sensorineural hearing loss (35–45 dB HL) in 46% of patients and severe sensorineural hearing loss (>74 dB HL) in 54%. The audiometric curve produced was flat in 30%, ascending in 27%, and descending in 43%. The healthy ear had normal hearing in 44 patients, mild hearing loss in 10 cases (25–34 dB HL), and moderate sensorineural hearing loss in one. The tympanogram was normal in all cases.

### 2.2. Methods

Otorhinolaryngological and neurological examination, audiometry, tympanogram, haemogram, and cranial magnetic resonance (T1 and T2 phases and FLAIR—fluid attenuated inversion recovery). The haemogram was performed in the emergency department before the patient started the corticosteroid treatment.

### 2.3. Statistics

A descriptive statistical analysis of the data, expressed as mean and standard deviation were carried out. Mann-Whitney *U* tests using Spearman's correlation coefficient (Rho) using IBM SPSS software, version 19, were performed.

### 2.4. Ethical Considerations

This study was performed in accordance with the principles of the Helsinki Declaration (1975, 1983). The research protocol, the patient information sheet, and the signed informed consent documents were all approved by the hospital ethics committee.

## 3. Results

### 3.1. Haemogram

**Figure 1:**
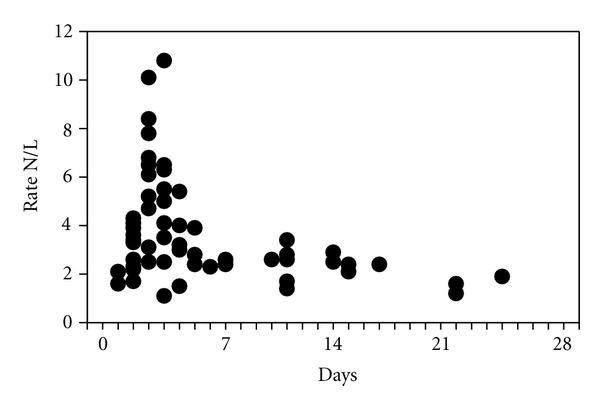
Individual values for the neutrophil/lymphocyte ratio (N/L) in patients with idiopathic sudden deafness (*n* = 41). Each point corresponds to the day on which the patient sought emergency treatment. Day 0 indicates the appearance of sudden deafness. The dotted line shows normal values. (Spearman's correlation coefficient (Rho) = −346; *P* = .009).

**Figure 2:**
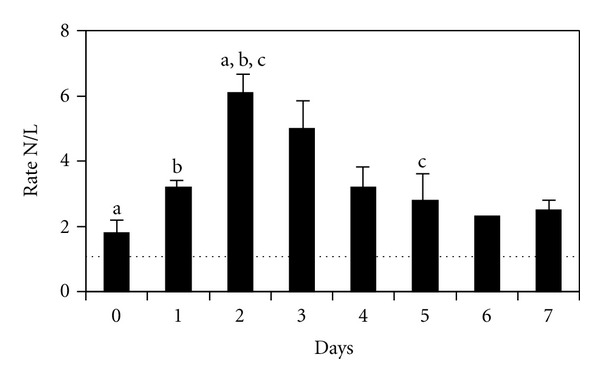
Mean ± standard error for the neutrophil/lymphocyte ratio (N/L) in patients with sudden deafness during the first week. Day 0 indicates the appearance of sudden deafness. The dotted line shows normal values. (Mann-Whitney *U* test. 0 versus 2: *P* = .032. 1 versus 2: *P* = .005. 2 versus 5: *P* = .043).

The acute-phase response in the peripheral bloodstream due to the inflammatory process caused by the transient cerebral ischaemia is manifested in the change of white blood cells. The neutrophil/lymphocyte ratio was determined.  Figure 1 shows the individual values of this ratio for the 54 patients.  Figure 2 displays the mean values for this ratio during the first week following the appearance of the sudden deafness.

### 3.2. The Neurological Examination, Including a Magnetic Resonance, Was Normal in All Cases

No significant differences between the values for the neutrophil/lymphocyte ratio and sex, age, initial hearing loss, or type of audiometric curve were observed. The other values of white blood cells and red cells were within normal limits.

## 4. Discussion

Sudden deafness was first described as a disease by De Kleyn [19] in 1944. It is defined as a sudden sensorineural hearing loss of at least 30 dB and at least three consecutive frequencies, normally in one ear, with no previous background of ear disease.

The causes of sudden deafness have been sought within the ear, with different proposed aetiologies such as a ruptured cochlear membrane, microangiopathic ear processes, viral ear infection, autoimmune diseases of the inner ear, Ménière disease, or vestibular schwannoma [1], although none of these theories has been sufficiently proven or can be applied in all cases. The aetiology for this condition is defined in only 10% of cases, whereas the rest are labelled as idiopathic. In young patients (younger than 50), it is usually associated with viral problems, where the most relevant viruses include measles, mumps, herpes zoster, and adenovirus [20]. After the age of 50 years, vascular disorders are the most common causes of this syndrome. The viral infections are characterised by decreased neutrophil levels and higher concentrations of mononuclear cells, such as lymphocytes and monocytes, which would reduce the neutrophil/lymphocyte ratio far below normal values [21], ruling out a viral origin of sudden deafness.

The incidence of sudden deafness has increased over time, and this condition has been estimated to reach 160 cases per year per 100,000 inhabitants [22]. The diagnosis is made based on clinical symptoms, audiometry, and a magnetic resonance that includes the internal auditory canals where the auditory nerve passes. The diagnosis of sudden deafness is made when the neurological examination is normal. 

Ischaemic processes of the inner ear have begun to be considered the pathogenic mechanism involved in sudden deafness [23]. Several publications have reported that patients diagnosed with idiopathic sudden deafness have a higher probability of suffering a stroke in the following 5 years [24, 25]. Genetic polymorphisms have been associated with inflammatory pathways in patients with sudden deafness [13, 14, 16]. 

Under ischaemic conditions in the central nervous system, an inflammatory response is produced which attempts to improve the hypoxia. In the peripheral bloodstream, the inflammatory reaction presents an acute-phase response, increasing the concentrations of neutrophils and monocytes and decreasing lymphocytes [7–10]. The objective measurement of increased neutrophil/lymphocyte ratio values during the transient ischaemic process, and its subsequent normalisation, provides evidence that sudden deafness is associated with inflammatory processes secondary to transient cerebral ischaemia, that is to say a temporal link exists, which was corroborated by our statistical analysis (Figure 2). The magnetic resonance images show the absence of organ damage, as in transient ischemic attacks. The normal neurological findings also corroborate this conclusion.

The acute-phase response reached its greatest intensity 48–72 hours following the appearance of the sudden deafness. In all cases studied, any other process that could have caused an inflammatory reaction was ruled out using the patient's clinical history.

The acute-phase response caused by the inflammatory process of the transient cerebral ischaemia is properly treated with corticosteroids, in compliance with the protocol for sudden deafness. Perhaps the most important result of acknowledging the presence of cerebral ischaemia in the development of sudden deafness is the severity of the process or, at least, the warning of the need to reexamine the patient for maintenance or advice by cardiology and then neurology specialists once again. 

The initial treatment of sudden deafness should be decided upon by the health professional that examines the patient, which would be an otorhinolaryngologist, provided the pathogenesis of cerebral ischaemia or any other organ disorder. The patient would then be referred to other specialists for the appropriate examinations.

 These study findings relate to a nonspecific systemic inflammatory response in sudden deafness, which actually aligns with recent findings in this area [11–18].

## 5. Conclusions

This study shows that there is an acute-phase response in the peripheral bloodstream with an increased neutrophil/lymphocyte ratio as an indirect evidence of an inflammatory process caused by transient cerebral ischaemia in sudden deafness. This same increase in the neutrophil/lymphocyte ratio also serves to rule out a viral origin for sudden deafness, since viral infections decrease neutrophil concentrations and increase lymphocyte counts, thus reducing the neutrophil/lymphocyte ratio. An objective measurement of these values aids in understanding the pathogenic mechanisms associated with sudden deafness, as well as in providing better care to the patients.
